# Commentary: Takotsubo Cardiomyopathy-Acute Cardiac Dysfunction Associated With Neurological and Psychiatric Disorders

**DOI:** 10.3389/fneur.2019.01163

**Published:** 2019-10-31

**Authors:** Josef Finsterer, Claudia Stöllberger

**Affiliations:** Krankenanstalt Rudolfstiftung, Messerli Institute, Vienna, Austria

**Keywords:** Takotsubo, stunned myocardium, peripheral nervous system, heart failure, central nervous system

With interest we read the review article by Buchmann et al. about Takotsubo cardiomyopathy (TTC), an acute reversible heart failure syndrome, triggered by neurological or psychiatric disorders (1). The authors concluded that the international expert consensus document on TTC, published by Ghadri et al. (2), “should be used in the daily clinical routine to provide excellent patient care” (1). We have the following comments and concerns.

We do not agree with the notion that only disorders of the central nervous system (CNS) cause TTC (1). Also disorders of the peripheral nervous system (PNS) have been shown to trigger TTC [Table 1; (3)] although these data are mainly provided by single case reports. For example, it is well-established that patients with myasthenia gravis who experience an acute myasthenic or cholinergic crisis leading to respiratory distress, can develop TTC (3). A myasthenic or cholinergic crisis may evoke fear of dying from respiratory insufficiency and the resulting catecholamine storm is believed to trigger TTC. A combined CNS/PNS disorder which has been reported to trigger TTC is amyotrophic lateral sclerosis (ALS) (4). ALS is characterized by rapidly progressive muscle weakness including bulbar and axial muscles, either already at onset of the disease or during the further course, and clinically manifesting as respiratory insufficiency or swallowing dysfunction. Furthermore, these patients may be confronted with the situation to suffocate, which may trigger the catecholamine storm (4). A further PNS disease reported to be involved in the development of TTC, is autonomic neuropathy in patients with diabetes (5). TTC has been also reported in hereditary motor and sensory neuropathy (HMSN) (6).

**Table 1:** Neurological and psychiatric conditions triggering TTC.

**Neurological/psychiatric triggers of TTC**	**Frequency**	**Level of evidence**
**NEUROLOGICAL TRIGGERS**		
Subarachnoid bleeding	Frequent	Studies
Seizures	Frequent	Studies
Intra-cerebral bleeding	Frequent	Studies
Ischemic stroke	Frequent	Studies
Meningitis/encephalitis	Rare	Studies
Transient global amnesia	Rare	Studies
Migraine	Rare	Studies
Traumatic brain injury	Rare	Case reports
Parkinsonism	Rare	Case reports
Brain tumor	Rare	Case reports
Dementia	Rare	Case reports
Multiple sclerosis	Rare	Case reports
Serotonin syndrome	Rare	Case reports
Aterio-venous fistula	Rare	Case report
Cyclic vomiting syndrome	Rare	Case report
Cerebral hypoxia	Rare	Case report
Acute myelitis	Rare	Case report
Hydrocephalus	Rare	Case reports
Chiari-I malformation	Rare	Case report
PRES	Rare	Case reports
**PSYCHIATRIC TRIGGERS**		
Affection disorder	Frequent	Studies
Anxiety disorder	Frequent	Studies
Psychosis	Rare	Case report
Substance abuse	Rare	Case report
Attention deficit hyperactivity disorder	Rare	Case report
Anorexia nervosa	Rare	Case report

*PRES, posterior reversible encephalopathy syndrome*.

We also do not agree that TTC may be triggered only by the CNS disorders subarachnoid bleeding, epilepsy, intracerebral bleeding, ischemic stroke, meningitis/encephalitis, migraine, or traumatic brain injury (1). CNS disorders other than those mentioned in the review being associated with TTC include Parkinsonism, brain tumors (7), dementia (8), multiple sclerosis, serotonin syndrome, and others (Table 1). In a review about TTC and neurological disorders these other CNS conditions need to be discussed. However, for several of these CNS conditions not included in the review by Buchmann et al. (1) data mainly derived from single case reports (9).

Concerning psychiatric disease as a trigger of TTC, not only affective disorders and anxiety disorders may trigger TTC but also in psychosis and substance abuse, particularly opiate withdrawal (10). Whether truly all TTC patients have high illness-related anxiety levels remains speculative.

The review lacks a discussion about echocardiography and cardiac MRI as a tool to diagnose TTC. Echocardiography is the imaging method of choice for detecting TTC in the acute situation. If the patient is in a stable condition and in case of uncertainty, ventriculography, or cardiac MRI may be useful alternatives, if available and applicable.

The review also does not extensively discuss the subtypes of TTC (apical, midventricular, basal, lateral, and global). Particularly, there is no discussion about the frequency of these subtypes among neurologically ill patients in relation to the triggering neurological disease.

Concerning the diagnostic criteria, it is crucial that coronary heart disease is excluded by coronary angiography or by CT-angiography of the coronary vessels.

Overall, the review by Buchmann et al. (1) has a number of shortcomings. The review does not mention PNS disorders as triggers of TTC. Also a number of CNS disorders which can trigger TTC has not been mentioned. Cardiac MRI is not discussed as diagnostic tool and the different subtypes of TTC were not considered.

## Author Contributions

JF: design, literature search, discussion, first draft, and critical comments. CS: discussion and critical comments.

### Conflict of Interest

The authors declare that the research was conducted in the absence of any commercial or financial relationships that could be construed as a potential conflict of interest.
